# Designing health information technology tools for behavioural health clinicians integrated within US-based primary care teams

**DOI:** 10.14236/jhi.v25i3.998

**Published:** 2018-10-31

**Authors:** Tanisha Tate Woodson, Rose Gunn, Khaya D. Clark, Bijal A. Balasubramanian, Katelyn K. Jetelina, Brianna Muller, Benjamin F. Miller, Timothy E. Burdick, Deborah J. Cohen

**Affiliations:** Department of Family Medicine, Oregon Health & Science University, Portland, OR, USA; Department of Family Medicine, Oregon Health & Science University, Portland, OR, USA; Department of Family Medicine, Oregon Health & Science University, Portland, OR, USA; Department of Epidemiology, Human Genetics, and Environmental Sciences, University of Texas School of Public Health-Dallas Campus, Dallas, TX, USA; Department of Epidemiology, Human Genetics, and Environmental Sciences, University of Texas School of Public Health-Dallas Campus, Dallas, TX, USA; Department of Family Medicine, Oregon Health & Science University, Portland, OR, USA; Eugene S. Farley, Jr., Health Policy Center, Department of Family Medicine, University of Colorado School of Medicine, Denver, CO, USA; Department of Community & Family Medicine, Geisel School of Medicine at Dartmouth, Hanover, NH, USA, Department of Biomedical Data Science, Geisel School of Medicine at Dartmouth, Hanover, NH, USA and Department of Medical Informatics & Clinical Epidemiology, OHSU School of Medicine, Portland, OR, USA; Department of Family Medicine, Oregon Health & Science University, Portland, OR, USA

**Keywords:** qualitative research, integrated care, primary care, electronic health records, behavioural health clinicians

## Abstract

**Background:**

Electronic health records (EHRs) are a key tool for primary care practice. However, the EHR functionality is not keeping pace with the evolving informational and decision-support needs of behavioural health clinicians (BHCs) working on integrated teams.

**Objective:**

Describe the workflows and tasks of integrated BHCs working with adult patients identify their health information technology (health IT) needs and develop EHR tools to address them.

**Method:**

A mixed-methods, comparative case study of six community health centres (CHCs) in Oregon, each with at least one BHC integrated into their primary care team. We observed clinical work and conducted interviews to understand workflows and clinical tasks, aiming to identify how effectively current EHRs supported integrated care delivery, including transitions, documentation, information sharing and decision-making. We analysed these data and employed a user-centred design process to develop EHR tools addressing the identified needs.

**Results:**

BHCs used the primary care EHR for documentation and communication with other team members, but the EHR lacked the functionality to fully support integrated care. Needs include the ability to: (1) automate and track paper-based screening; (2) document behavioural health history; (3) access patient social and medical history relevant to behavioural health issues and (4) rapidly document and track progress on goals. To meet these needs, we engaged users and developed a set of EHR tools called the Behavioural Health e-Suite (BH e-Suite).

**Conclusion:**

US-based integrated primary care teams, and particularly BHCs working with adult populations, have unique information needs, workflows and tasks. These needs can be met and supported by the EHR with a moderate level of modification.

## INTRODUCTION

**Figure 1:**
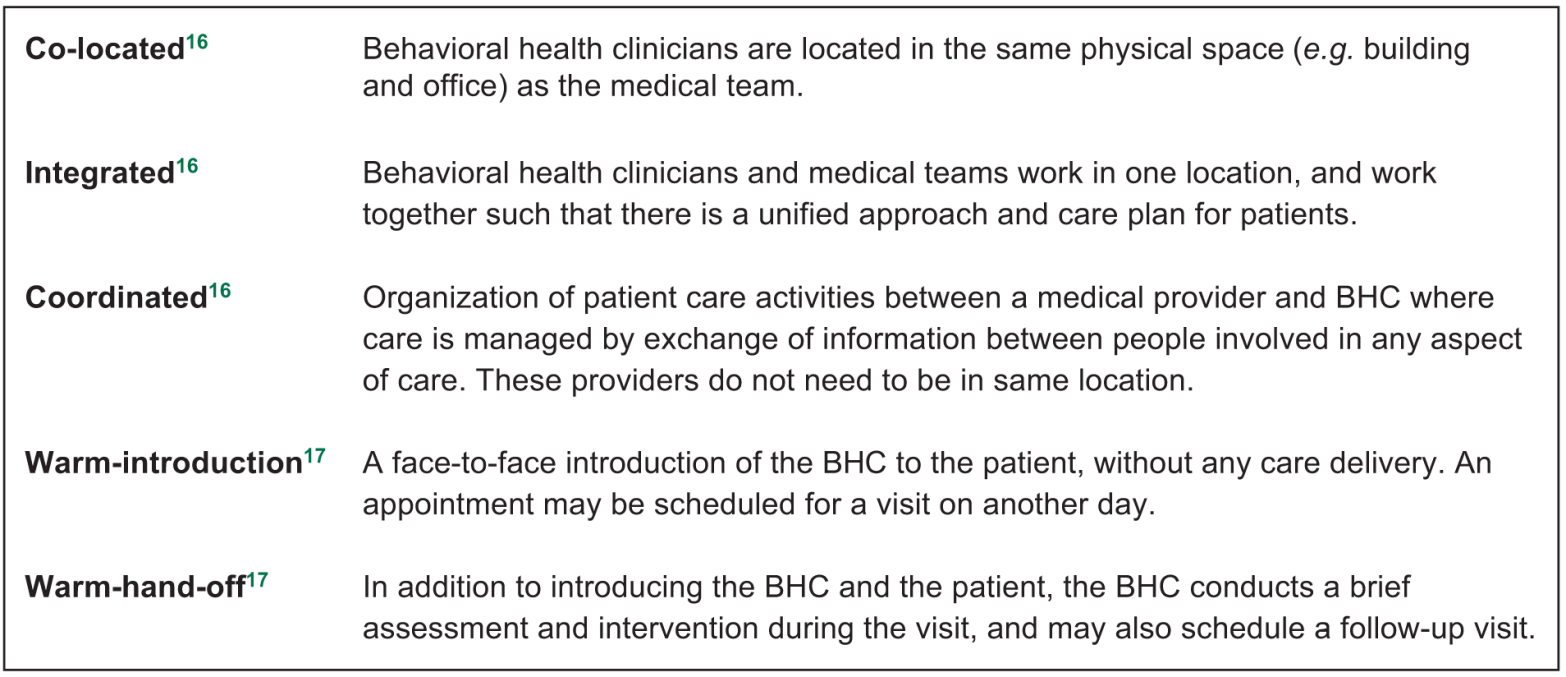
Definitions of integration approaches.

The integration of behavioural health and medical care brings benefits to individuals and health systems,^1,2^ including improved care quality, patient experience, physician satisfaction and reduced healthcare costs.^3-6^ Primary care practices are including behavioural health clinicians (BHCs) on their clinical teams to provide whole-person integrated care.^1,7-10^ BHCs are typically licensed clinical social workers or clinical psychologists that work in primary care practices with adult patients to provide brief therapy to address mild to moderate behavioural health needs such as substance abuse, health behaviours, life stressors and other crises.^11^ Various approaches to include behavioural health in primary care use the terms ‘colocation’, ‘integration’ and ‘coordination’.^12-15^ While we use Peek’s definitions^11^ to distinguish these approaches (see Figure 1), for this study, we use ‘integration’ to refer to *any* approach a practice uses to bring behavioural and medical care together in one location, and ‘behavioural health’ to encompass mental health, substance use and other behaviours that might influence physical health.^15^

Electronic health records (EHRs) are a key tool for primary care practice. However, EHR functionality is not keeping pace with the evolving informational and decision-support needs of BHCs working on integrated teams.^18^ This inability of EHRs to support delivery of integrated care has emerged both in response to policy decisions (BHCs were excluded from being considered eligible providers for meaningful use incentive payments), but also from the distinction and separation of the behavioural health and primary care cultures, leading to the creation of entirely unique products for these disciplines. EHR vendors and healthcare organisations focused development efforts elsewhere, and behavioural health had to address their information needs on their own.^19-21^

While findings are mixed regarding the benefits of the EHR use in primary care settings,^22-26^ it is evident that the quality and utility of an EHR rest on the tool’s alignment with the workflows, tasks and cognitive processes of users.^26^ While EHRs can be adapted to support the workflow, task, informational and decision-support needs of integrated teams, few have examined these needs and how EHR redesign might address them. We describe these integrated team needs with the aim of identifying and addressing them through the development of an innovative EHR tool suite called Behaviour Health e-Suite (BH e-Suite).

## METHODS

### Design and sample

We purposively selected six federally qualified health centres (FQHCs) in Oregon to participate in a mixed method, observational, comparative-case study called turning EHRs into Assets for Mental Health and Uniting Practice (TEAM-UP) funded by the National Institute of Mental Health (1 R34 MH100371-01). Practices were members of OCHIN, Inc., a network of community health centres (CHCs) using a shared license of Epic™ (Epic Systems Corp., Verona, WI).^27,28^ Practices used OCHIN Epic for 2–5 years, employed at least one BHC at the time of study recruitment, and were early (2 years or less) into implementing an integration approach. We purposively selected practices that varied with regard to the geographic location (urban, rural and suburban), size and approach to integration (integration, co-location and coordination, see Table 1). We expected these attributes would influence integrated team workflows, information needs and tasks, and we wanted to ensure that the EHR tools we developed would be usable by a wide range of practices.

For the purposes of this paper, we adopt the terminology used among the practices in this sample, referring to a BHC as a care team member who delivers brief, problem-focused therapy to patients. The term mental health clinician (MHC) is used to reference professionals, often co-located in the practice, who are employees of a community mental health centre (with various degree types) and deliver long-term, traditional therapy to patients.^29^

### Data collection

The data-collection team was composed of researchers experienced in qualitative, primary care delivery, informatics and human factors research. We conducted site visits between November 2013 and May 2014. Length of site visit varied between 2 and 4 days depending on the practice size, and focused on intensively observing the EHR use by the integrated care team. We observed primary care clinicians (PCCs), BHCs, medical assistants (MAs), front desk staff, MHCs and other key members of the clinical care team; this included observing the preparation and completion of visits, and their interactions with patients when permitted. We also observed individual work areas and team work areas. During each site visit, we conducted semi-structured interviews, following a guide (see Appendix A) tailored to both the practice and the interviewee. We conducted interviews with two-to-four practice members representing different roles in the practice (*e.g.* Medical Director, BHC, MHC, PCC and MA) to understand the specific needs and workflows of integrated care teams.

### Data management

We took annotated field notes on site and prepared detailed notes describing observations, within 24–48 hours of the site visit. We audio-recorded and professionally transcribed interviews, and checked the transcripts for accuracy. Field notes and interview transcripts were de-identified and entered into Atlas.ti (Version 7.0, Atlas.ti Scientific Software Development GmbH, Berlin, Germany).

### Analysis

Our team analysed these data using an immersion-crystallisation approach^30,31^ in three steps. First, we reviewed the data collected from field notes and interviews at each practice to identify integrated teams’ workflows, tasks, information and EHR needs. Through this process, we created a codebook for labelling data,^32^ and used these codes in group analysis sessions until all members reliably defined and used codes the same way. After analysing each practice’s data, we analysed data a second time to make cross-practice comparisons. While PCCs and their team members did not identify specific integrated care informational needs to be addressed, BHCs identified a range of unaddressed documentation and informational needs. This discrepancy may be due to OCHIN’s history of making EHR adaptations to optimise primary care delivery. Thus, we focused attention on identifying the workflows, tasks and communication, information and documentation needs mentioned by BHCs.

Second, we developed workflow diagrams for each practice identifying the tasks and processes involving BHCs, case summaries identifying documentation and information needs, and ideas for how to improve EHR tools. Third, we examined findings across practices to identify common and disparate EHR needs. We shared these findings with developers and a BHC user group (six to eight members), and engaged in a user-centred design process to develop solutions. The result was the development of the BH e-Suite, a set of EHR tools customised to meet the informational needs of BHCs and integrated teams. For more on the tool development process, see Clark *et al*^33^

The Institutional Review Board at Oregon Health & Science University approved this study protocol (#9366).

## RESULTS

### BHC tasks and workflow

**Figure 2:**
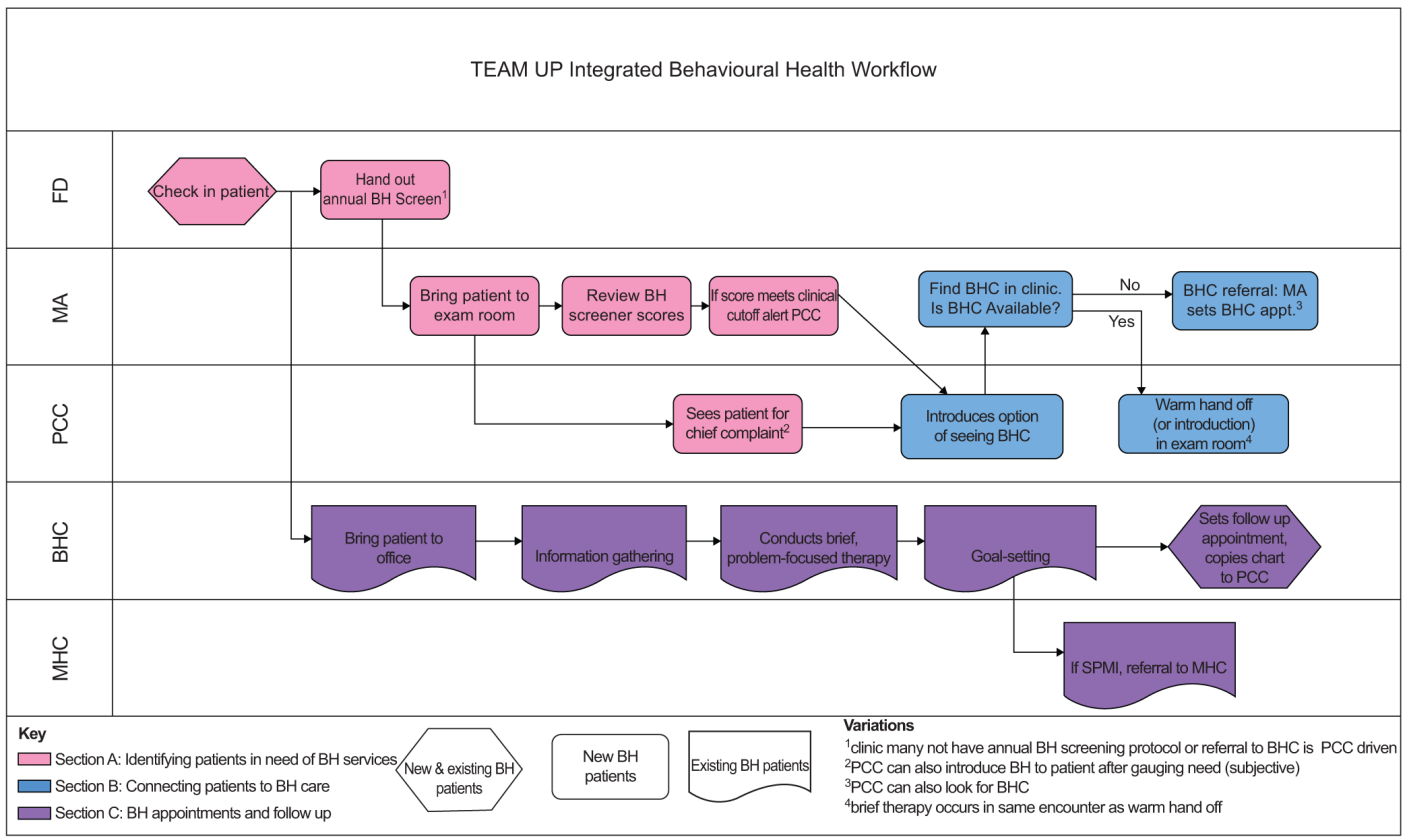
TEAM-UP integrated BHC workflow

Figure 2 shows the predominate workflow observed across the clinics for BHCs working on integrated care teams. We observed three steps in this process: (1) identify patients in need of behavioural health services; (2) connect patients to behavioural health care services and (3) follow up with patients that have a series of BHC appointments. Table 2 identifies three areas where health IT challenges were encountered. During the detection of patient behavioural health needs, we observed assessment and documentation challenges, and when patients were connected to behavioural health services, BHCs noted information retrieval issues. As BHC engaged in brief treatment with patients, they lacked tools to track the patient progress. Table 2 also shows the EHR functionality we developed to address these needs.

#### Identifying patients in need of behavioural health services

As shown in Figure 2, Section A (pink highlight), patients in the need of behavioural health services were identified in two ways: (1) through a systematic screening process, in which front desk staff provide patients with a paper-based screening tool that includes behavioural health assessments chosen by the practice (*e.g.* PHQ-2/9^34^ and GAD-7^35^), later ‘scored’ by the MA or (2) through a subjective assessment (*e.g.* patient discusses depression symptoms with doctor). In these cases, the medical team may not do a formal assessment, but the clinician determines if a behavioural health visit would be useful.

We found that paper-based assessment tools were entered into several areas of the EHR after being manually scored – the Questionnaires section, Assessments section and the clinician’s Progress Note – either by a medical team clinician, staff member or by the BHC. This process can be time-consuming, and requires calculating and recording cumulative assessment scores, and sometimes scanning and attaching the paper document to the EHR. Paper assessments could also be lost when patients transitioned from the PCC to the BHC, interfering with timely care delivery. BHCs expressed the need to reduce paper screening use, and voiced an interest in an EHR template for tracking patient scores over time. BHCs also wanted screening tools and scores to be in a single EHR location.

We addressed these needs by developing the BH assessment tab, which allows primary care team members to select common screeners from a drop-down list of options, and provides screening templates that auto-tabulate scores and track screening scores over time. Care team members can also review screening data over time in this location.

#### Connecting patients to behavioural healthcare

For patients wanting to visit a BHC, there were two workflows for making this connection (see Figure 2, Section B, blue highlight). On the first path, the MA or PCC would look for the BHC during the primary care visit, and if found, a warm introduction or warm handoff would happen. With both the warm handoff and warm introduction, there is verbal communication and a shared care element where both PCCs and BHCs are in the examination room talking with the patient.

On the second path, patients got connected to the BHC through a referral. The PCC copies the BHC on the patient’s encounter note and the PCC, MA or front desk staff schedules an appointment for the patient with the BHC. In some practices, this is how patients were always connected with BHCs; in others, this referral path was taken only when the BHC was unavailable.

Regardless of how BHCs and patients were connected, BHCs needed to easily retrieve patients’ chart information to develop an understanding of the patient’s history and needs. In warm handoffs, PCCs would verbally communicate this to the BHCs. With referrals, there was no verbal communication, and PCCs did not always enter the referral reason in the EHR referral order. When preparing for patient appointments, BHCs would search several areas of the medical chart (*e.g.* appointment notes, medical and behavioural health history fields) for the reason for the referral. BHCs expressed the need for an easier way to quickly access patients’ relevant social, medical and behavioural health history.

We developed two BH e-Suite features to address this: the personal and family BH history and snapshot. The personal and family BH history feature pre-populates the patient’s social history and their family’s BH history using frequently used terms, and provides a link to the patient’s medical history for quick review. The snapshot feature provides a summary of the relevant behavioural health information (*e.g.* BH history, diagnosis and record of BH screening scores) so no searching around is necessary.

#### BHC appointments with patient and follow-up

Once the patient information was gathered, BHCs delivered a problem-focused therapy and/or assistance, which could include teaching (*e.g.* mindfulness training), connecting patients with other resources (*e.g.* support groups) and goalsetting with the patient (Figure 2, Section C, purple highlight). Patients had one-to-six visit(s) with BHCs; during the visit, the BHC documentation involved free-text typing into the progress note. As BHCs wanted to build rapport and make eye contact with the patients, they would wait after visits to type progress notes. BHCs expressed the need to: have the ability to quickly select diagnosis codes and indicate severity; to quickly document behavioural health history during appointments and to have this information auto-populate progress notes and to have easy ‘point and click’ methods for populating eligible BH billing codes for reimbursement.

We developed three BH e-Suite features – visit diagnosis, progress note and level of service (LOS) – to address these BHC documentation needs. The visit diagnosis feature allows BHCs to select the appropriate diagnosis from a list of options and to make annotations regarding severity (*e.g.* mild, moderate and severe). The progress note provides speed buttons for quick documentation and auto-populates information from the BH assessment tab and the visit diagnosis feature. The LOS feature provides speed buttons configured with common BHC billing codes.

BHCs often had several follow-up visits with patients, and needed to quickly orient themselves with the patient (*i.e.* understanding assessments, recalling previous discussions with the patient and identifying goals the patient had set). We found that the current EHR did not allow BHCs to track patient scores on diagnostic tools over time, hindering BHCs’ ability to easily assess the patient progress. The EHR also did not include tools to document patients’ goals or challenges and strengths, and it did not have mechanisms to track these over time. BHCs also wanted the ability to send patients a followup message after a visit.

To address these needs, we developed the goals, challenges/opportunities feature to assist BHCs with documenting and reviewing patient goals, challenges and opportunities to change. The feature provides templates to quickly document and review common strengths and social barriers and provided a table to document goals using a drop-down list of common self-management goals. This section also provides a summary of the patient goals over time.

BHCs wanted tools to support the patient outreach and panel management (*e.g.* to generate a list of recent patients, and then to sort or filter that list based on the patient conditions). One tool included in the BH e-Suite is a dynamic reporting workbench report used to track any patients meeting the BHC’s criteria for follow-up. These might include patients not seen recently or those identified by PCCs as possibly benefitting from behavioural health services, but who have not yet engaged. To support the patient outreach, this report displays each patient on a separate row and includes pertinent clinical information such as the date of the last BHC appointment, the last PHQ-9 score and the other behavioural health assessment data.

## DISCUSSION

We learned through a process of discovery that BHCs on integrated primary care teams had unmet documentation and information needs, especially with regard to the integration of care. Adapting to the culture of primary care, BHCs on integrated teams worked at a pace similar to PCCs (brief and frequent visits); they aspired to document during or immediately following a visit and they preferred to use rapid and unobtrusive point-and-click documentation approaches during visits. BHCs needed more efficient tools for assessment, information gathering and documenting goals and challenges. They also needed notes in a format that was easily shared and reviewed by other team members. In collaboration with BHCs, informatics experts and developers, we developed the BH e-Suite. The BH e-Suite features quick buttons, dropdown menus and point-and-click fields to record information about care, as well as behavioural health screening tabs that automatically calculate scores and populate patient notes. Some of the tools in the BH e-Suite have subsequently been adopted by Epic Systems Corp. and released with newer versions of the EHR software.

Our study highlights the need for healthcare organisations considering investment in health IT as part of its transformation cost when transitioning to an integrated care approach. EHRs are common tools in primary care practices,^36,37^ and are critical to the delivery of integrated, team-based care.^38,39^ Off-the-shelf EHRs are not designed to support the work of integrated teams.^26^ Investment in EHR tailoring is necessary to facilitate the documentation and information sharing inherent to integration. Our study provides a starting point for considering EHR adaptations practices might need to make.

Investments in EHR modification can be costly, and having EHR vendors improve their systems through federal mandate would ensure that all practices have optimal EHR tools for integration. Our study suggests the need for EHR vendors and developers to recognise there are different ways of integrating patient care, and that these approaches may shape documentation and information-sharing requirements. While we identified a common set of informational, documentation and tracking needs for integrated teams, these needs can also vary within practices. Our findings show that workflows vary due to patient flow and time constraints. EHR systems need to be designed to support the teams’ needs under these varying circumstances.

## LIMITATIONS

This study has a number of important limitations. First, our small sample of practices was all CHCs or rural health clinics using a single EHR system. This was necessary to gain the depth of knowledge needed to inform development, and to have the resources and expertise necessary to tailor the EHR system. We mitigated this limitation by purposively selecting practices that varied in size, location and approach to integration, and by including BHCs not part of these clinics in the design efforts. While the solutions we developed are potentially unique to epic and limited in generalisability, the user needs we identified are likely common, as research shows that similar needs emerged among BHCs practicing in community and academic primary care settings.^29^ Thus, our findings may be widely applicable and could inform the design efforts of non-epic EHR systems; more research is required to make this determination.

Second, at the time of the study, BHCs had no federal or state documentation or billing requirements, and insurers were not paying BHCs for integrated services. We may have found more commonality among BHC billing documentation needs because state and federal requirements were not yet driving their work. Healthcare organisations and developers will need to be cognizant of these requirements, as this will shape the EHR development, and finding ways to make documentation for billing purposes easier will be important. Third, we did not assess the acceptability of ease of using the BH e-Suite tools we developed. Future research is needed to conduct such an assessment, and to examine the effect of BH e-Suite use has on process and outcome measures relevant to integrated care.

## CONCLUSION

The pace of change in the primary care setting is rapid. To achieve the triple aim and to transform into a patient-centred primary care home, practices are expanding their primary care team to include new professionals (*e.g.* BHCs) and changing how patient care is delivered. Addressing the new documentation and information needs of multi-disciplinary care teams is crucial to their success. We identify a number of unmet EHR needs of BHCs working on integrated primary care teams,^40-42^ and describe real-world solutions for addressing these needs. As such, these findings can be a helpful starting place for vendors and healthcare organisations refining their EHRs to better meet the needs of integrated teams.

## Figures and Tables

**Table 1:** Characteristics of the six participating practices

	Practice 1	Practice 2	Practice 3	Practice 4	Practice 5	Practice 6
Ownership	FQHC	FQHC	FQHC	FQHC	FQHC/CMHC	FQHC
Location	Rural	Urban	Rural	Suburban	Urban	Urban
Active patients	2103	5338	4931	5306	3021	938
BHC employee	Internal	Internal	External CMHC	External CMHC	-	Internal, BHC is grant-funded position
BHC *n*	1	2	1	1	-*	1
MHC *n*	-	1	1	1	17	-
Inception of BHC integration^†^	6 months	2 years	2 years	2 ½ years	-	3 months
Integration approach	Integrated	Integrated	Integrated/coordinated	Coordinated	Co-located	Integrated
FTE ratio BHC:PCC	1:9	2:16	1:7	1:20	-	1:2

BHC = behavioural health clinician; MHC = mental health clinician; PCC = primary care clinician; FTE = full-time equivalent.

* This practice had a BHC that left after recruitment and never replaced them. This clinic had 17 MHCs and two PCC which provide feedback on the BH e-Suite tool

† Inception is calculated from the start of integration at the practice to the start of our study.

**Table 2:** Characteristics of the six participating practices

BHC health IT challenge/need	Feature developed	Description of new BH e-Suite feature
Assessment and documentation needs		
Reduce use of paper screening tools	BH assessment tab	Drop-down lists and templates of common BHC screeners build into EHR
Eliminate manual entry and tabulation of scores		Templates auto-tabulate scores
Track scores over time		Screening scores displayed over time (date of screening noted) with an indication of risk level
Quickly select diagnosis codes and indicate the severity	Visit diagnosis	A drop-down list of diagnosis tailored to the BHC Diagnosis can be annotated as mild, moderate or severe
Quickly document patient progress during appointments	Progress note	Speed buttons and free-text comment fields provided on assessment page for quick documentation All of the features described above auto-populated progress note; BHC retain the ability to add free-text notes, as needed
Populate eligible BH billing codes for successful reimbursement	Level of service (LOS)	Speed buttons configured with LOS codes used by BHCs for billing purposes; LOS codes customised to include billable BHC encounters (Oregon only)
Information retrieval needs		
Quickly identify personal and family history related to BH	(1) Personal and Family BH history (2) Snapshot	Pre-populate family history tailored for BHCs with commonly used information such as history of anxiety, depression, substance use, diabetes, high cholesterol and blood pressure
Quickly review BH history during a visit		Pre-populate patient social history using frequently used terms, including head trauma, insomnia, sexual abuse and trauma. Provide a link to medical history for quick review Summary section populated with BH relevant information (BH history, diagnosis and record of BH screening scores)
Monitoring and tracking needs		
Mechanism to easily document goals	Goals, challenges/opportunities and follow-up	Table created to document goals with a drop-down list of common self-management goals
Document/review patients challenges and opportunities to change/achieve goals		Template developed to quickly document/review common strengths and social barriers and comment box for elaboration on details
Track patient goals over time		The summary section that provides a snapshot of the patient goals over time
Track patient panel for follow-up and outreach		Lists details to review for the patient’s next visit
	Repurposed existing space and tools – Reporting Workbench	Communicate scheduling instructions for Front Desk staff Ability to forward the encounter with a message to another clinical staff member Reminders function created in In Basket; BHC uses this to recall specific tasks.

## APPENDIX A

### TEAM UP Study – Pre-Site Visit Interview

This phone interview will be conducted with the site liaison and other key practice members who were identified as key informants. This interview will be conducted prior to visiting the site, and can be viewed as both a planning call and a tool for collecting data about the practice. This interview will help plan the site visit, tailor observation and interviewing tools, and allow us to be as efficient as possible when on site. To that end, this pre-site visit interview will help us identify key informants to interview at the practice, and we will schedule those interviews in advance of the site visit.

Please Note: Ask for permission to tape record this interview.

Thank you so much for joining us today. We are excited to be planning a site visit to your exceptional clinic. We would like to take a moment to have everyone on the call introduce themselves.

Thank you so much for completing the Practice Information Form, which has really helped us understand some of the demographic or organisational characteristics of your practice. Today, we would like to know more about the operational characteristics regarding the delivery of integrated care in your clinic. This will help us understand your practice better and plan our visit with you.

1. Tell us about how your practice is organised?
  Possible probes
  - Tell me about how the practice handles scheduling visits.
  - Does the office operate in teams or pods?
  - What is the allotted time for the different types of visits your practice offers (well care, behavioural health, acute, chronic and chronic follow-up)?
  - Who are the people ‘in charge’?
  - What days are behavioural health providers available? About how many patients do they see a day?
  - Do patient care teams meet or huddle either before or during patient care sessions?
  - Think about the physical layout of the practice, where do clinicians and other healthcare professional talk to each other during the patient care process?
2. Please assume that we know very little about how your practice delivers integrated care. Can you walk us through the process of delivering integrated care?
  Possible probes
  - Can you describe the check-in process?
  - How do people know what type of visit the patient is coming in for?
  - Can you describe when and how screening is done?
  - How are referrals handled for behavioural/mental health services? Who is involved in this process? What do these people do?
  - What happens after a referral is made?
    - How do you track follow-through on referrals?
    - What the communication between clinicians and the behavioural/mental health providers they refer to?
    - How do you monitor the patients who are improving?
    - What do clinicians do if the patients are not improving?
3. How has your practice changed the workflow to accommodate the integrating behaviour health?
  Possible probes
  - How has collaboration changed?
4. Can you tell me about how the Epic EHR and BH navigator are used in the practice?
  Possible probes
  - Who are the ‘super users’ in the practice, the people that use this tool the most often are the most proficient?
  - How were practice members trained in using this tool?
  - How are gaps in knowledge addressed? Who fills those gaps?
  We would like to interview people from your practices that are ‘experts’ in how your practice operates and delivers integrated care. We like to interview people with a range of expertise, and people who use the BH Navigator.
5. Can you identify practice members who are experts in how your practice functions to deliver integrated care?
  Possible probes
  - Which clinicians do you think we should interview?
    - Physical health
    - Behavioural health
  - Which medical assistant do you recommend us to interview?
  - Other nurses
  - Care manager or care coordinator, if they have one
  - Biller
  - Front desk staff
  - Office Manager
  - Information technology

### TEAM UP – Interview Guide

The questions below are the general topic areas we will explore with interview participants. These questions will be modified in light of what is learned during practice observation and to fit the expertise of the interviewee.

### Opening

Thank you for participating in this interview. We are talking with you today because we are interested in your experiences with delivering healthcare to patients in this practice. During the interview, I will ask you to tell me a little bit about yourself and your thoughts and experiences with the work that you do in this practice, and your use of the electronic health record system.

1. First, Please tell me about yourself?
  Possible probes
  - Educational background
  - Role in clinic
  - Prior work experience – how came to be working at this clinic
  - Other experiences
2. Please tell me what ‘integrated care’ means to you?
3. Please tell me about your experience using your EHR in delivering integrated care?
  Possible probes
  - Can you tell me about the ways the EHR supports delivering integrated care?
  - Can you tell me about the ways the EHR is a barrier to deliver integrated care?
  - If you could wish for some new EHR features to support you in delivering integrated care, what would they be?
4. Please think about a typical workday at [insert name of practice]. Walk me through your average day?
  Possible probes
  - Tell me more about the role you play in that?
  - Who do you work with to do that [fill-in]?
5. Now, please think about the tasks that you do in a typical day. Can you describe for me how you use the BH navigator in these tasks?
  [This series of questions about tasks may require moving to a computer for the person to show how the BH Navigator is used in each task.]
  Possible probes
  - In what ways is the BH Navigator useful in accomplishing this task?
  - In what ways might the BH Navigator be improved to help you accomplish this task more completely or efficiently?
6. Now, please think about the BH Navigator utilities. In what ways has using this system changed your work?
  Possible probes
  - Performance changes
  - Productivity changes
  - Efficiency changes
  - Communication changes
  - Collaboration changes
  - Information sharing changes
7. Please tell me about your training for the EHR system and the BH navigator?
  Possible probes
  - What do you feel was a really effective part of your training?
  - What do you feel could be improved?

Thank you so much for taking the time to speak with me today. We greatly appreciate it.
